# Lycium barbarum polysaccharide (LBP) inhibits palmitic acid (PA)-induced MC3T3-E1 cell apoptosis by regulating miR-200b-3p/*Chrdl1*/PPARγ

**DOI:** 10.29219/fnr.v64.4208

**Published:** 2020-12-22

**Authors:** Lei Jing, Baiwen Hu, Qing Hua Song

**Affiliations:** 1 Orthopedics Department, Ningbo First Hospital, Ningbo City, Zhejiang Province, China; 2 Plastic Surgery Center and Trauma Center, Ningbo First Hospital, Ningbo City, China

**Keywords:** LBPs, PA, osteoblasts, miR-200b-3p/Chrdl1/PPARγ, apoptosis

## Abstract

**Background:**

Obesity is closely related to osteoporosis. Lycium barbarum polysaccharides (LBPs) have anti-osteoporosis activity.

**Objective:**

This study aimed to explore the role of LBPs in palmitic acid (PA)-induced osteoblast apoptosis.

**Methods:**

The microarray data set *GSE37676* was downloaded from Gene Expression Ominibus (GEO) database. Top 300 differentially expressed genes (DEGs) were used to construct a protein–protein interaction (PPI) network based on STRING database, and significant modules were analyzed and their key genes were screened by using Cytoscape software. COEXPEDIA database showed that there was co-expression between *Chrdl1* and peroxisome proliferator-activated receptor (PPARγ). MC3T3-E1 cells were treated with 100–500 μg/mL of PA. Reverse transcription polymerase chain reaction (RT-PCR) and western blot assays were used to detect mRNA and protein levels. Cell Counting Kit-8 (CCK-8) assay and flow cytometry were used to detect cell viability and cell apoptosis.

**Results:**

*Chrdl1* was the key gene from the most significant module and downregulation in MC3T3-E1 cells treated with PA. MicroRNA miR-200b-3p and PPARγ were significantly upregulated among PA-treated MC3T3-E1 cells. The results of luciferase reporter gene assay showed that miR-200b-3p targeted *Chrdl1 3’-UTR*. Over-expressing miR-200b-3p promoted PA-induced cell apoptosis and inhibited cell viability. After pre-treating cells with PA and LBP, MC3T3-E1 cell apoptosis rate was relatively lower than that of mimics+PA_200_ group. *Chrdl1* inhibition partly reversed miR-200b-3p effect on inhibiting apoptosis among MC3T3-E1 cells pre-treated with LBP and PA. Decreased C CASP3, PPARγ and increased *Chrdl1* by miR-200b-3p inhibition were partly reversed by *Chrdl1* inhibition.

**Conclusions:**

LBPs inhibit PA-induced MC3T3-E1 cell apoptosis by mainly decreasing miR-200b-3p to upregulate *Chrdl1*, but miR-200b-3p/*Chrdl1*/PPARγ is not the only mechanism for LBPs protecting osteoblasts from PA.

## Popular scientific summary

- LBP suppresses the PA-induced osteoblast apoptosis via the miR-200b-3p/Chrdl1/PPARγ axis to play a protective role for obesity-induced osteoporosis, but miR-200b-3p/Chrdl1/PPARγ is not the only mechanism for LBPs protecting osteoblasts from PA.

Two metabolic diseases that have attracted global attention at present are obesity and osteoporosis; both have severely affected the health, qualify of life, and working competence of patients (1). Research suggests that obesity is closely related to osteopenia (2). Osteoporosis is a type of systemic bone disease featured by decline in bone mineral density (BMD) and destruction of bone tissue microstructure (3). Various bone growth factors, pro-inflammatory cytokines, and hormones bind with its receptor to activate various transcription factors that promote the survival, maturation, and differentiation of osteoblasts, thus exerting a variety of physiological effects (4). Therefore, osteoblasts have an important part during osteogenesis. Obesity induces increased adipocyte differentiation, lipid accumulation, and reduced osteoblasts differentiation, thereby resulting in decreased bone formation (5). Moreover, research has indicated that obesity can change the expression of multiple signaling pathways involving bone apoptosis, bone formation, bone differentiation, and bone adsorption (6). In our experiment, we simulated the *in vivo* hyperlipidemia environment of osteoblasts through palmitic acid (PA) treatment *in vitro* to further examine the relationship between obesity and osteoporosis.

As a traditional valuable herbal medicine in China, fructus lycii is a mature fruit of Solanaceae *Lycium barbarum*, which is mainly produced in Ningxia, Gansu, and Sinkiang. *Lycium barbarum* polysaccharide (LBP), one of the main active ingredients in fructus lycii, is suggested in pharmacological experiments to have anti-aging, anti-hyperlipidemia, obesity-improving, anti-fatigue, anti-tumor, anti-oxidation, and BMD-increasing effects (7, 8). In China, fructus lycii is frequently used to treat bone injuries. According to reports, LBP can upregulate osteoblasts activity to promote its growth; besides, it suppresses the activity of peroxisome proliferator-activated receptor (PPARγ) and reduces fat production to improve obesity (9, 10). Results of our previous research have demonstrated that LBP could suppress endoplasmic reticulum stress (ERS) to reduce the PA-induced osteoblast apoptosis (11). As it is well known, LBP is an active ingredient of traditional Chinese medicine, possessing multiple pharmacological molecular mechanisms. Therefore, this study aimed to further explore other molecular mechanisms by which LBP reduces the PA-induced osteoblast apoptosis.

MicroRNAs (miRNAs) have been recognized as single-stranded non-coding RNA molecules with the length of approximately 22 nucleotides and coded via endogenous genes. They have been extensively distributed in the eukaryotic cells of a variety of organisms. miRNAs can regulate cell growth, differentiation, and apoptosis to participate in the growth and development of organism as well as disease genesis and development (12, 13). It has been found in research that the abnormally expressed miRNAs can specifically regulate the expression of bone formation-related signaling pathways and transcription factors which are thereby closely correlated with osteoporosis genesis (14). miR-206 can suppress the osteoblasts differentiation and miR-26 can promote osteoblast differentiation (15, 16). Besides, miRNAs also exert indispensable role in the field of obesity. In thin and obese mice, the excessive intake of fructose elevated cholesterol and mRNA miR-27a levels, downregulated mRNA miR-33a level in the adipose tissues of liver, and elevated mRNA miR-21 level in plasma (17–19). miR-200b-3p is reported to inhibit breast cell proliferation meanwhile improving chondrocyte viability in osteoarthritis patients (20, 21); however, the miR-200b-3p role in obesity-induced osteoporosis has not been reported yet. Consequently, this study adopted bioinformatic analysis and cytological experimental techniques to explore miR-200b-3p function and its mechanism in PA-induced osteoblast apoptosis.

## Materials and methods

### Microarray data

Gene expression data, *GSE37676*, were provided by the Gene Expression Ominibus (GEO) (https://www.ncbi.nlm.nih.gov/geo/) database. Data from three samples of untreated MC3T3-E1 cells (GSM925429, GSM925430, and GSM925431), and from three samples of MC3T3-E1 cells treated with ascorbic acid (GSM925432, GSM1925433, and GSM925434) were obtained from the *GSE37676* dataset. In addition, differentially expressed genes (DEGs) were obtained using the GEO2R online approach.

### Construction of the protein–protein interaction (PPI) network as well as module analysis

The online annotation approach for biological function and localization of protein in cell, that is, Search Tool for the Retrieval of Interacting Genes (STRING), was utilized for predicting the PPI data. The top 300 DEGs were mapped into STRING to assess relationships of PPI; typically, the confidence value of >0.4 was regarded as significant. Cytoscape was utilized to visualize PPI-integrated networks. Then the MCODE plug-in of Cytoscape was employed to analyze modules within the as-constructed PPI network, and the following default parameters were determined, including ‘node score cutoff = 0.2’, ‘degree cutoff = 2’, ‘max depth = 100’, and ‘K-core = 2’.

### COEXPEDIA analyses

The *Chrdl1* co-expression was examined from COEXPEDIA (http://www.coexpedia.org/) database, the context-related co-expression network database constructed based on single mouse and human microarray sample series on the basis of GEO database.

### Cell culture

MC3T3-E1 cells were provided by Shanghai Cell Bank of the Chinese Academy of Sciences (Shanghai, China), which were cultured in α-MEM medium containing 10% fetal bovine serum (FBS; Gibco; Thermo Fisher Scientific Inc., Waltham, MA, USA) in an incubator under humidity, 5% CO_2_, and 37°C conditions.

### Cells treatment

Cell treatment could be divided as follows:

1. MC3T3-E1 cells were rinsed using PA of different contents (including 500, 400, 300, 200, 100, and 0 µg/mL) for a period of 12/24 /48 h.
2. Cells were subjected to 12 h of LBP pre-treatment at different contents (400 and 100 µg/mL). Then the medium was added with PA at 200 µg/mL for 12-h incubation.
3. Cells were then subjected to 12 h of PA pre-treatment at 200 µg/mL of PA prior to 48 h of miR-200b-3p mimics/inhibitors transfection or their negative control (NC; mimics/inhibitors control).
4. Cells were subjected to 24 h of LBP pre-treatment at different contents (400 and 100 µg/mL). Later the medium was added with PA at 200 µg/mL for 12-h incubation; then the cells were subjected to miR-200b-3p mimics/inhibitors transfection and negative control (mimics/inhibitors control) for 48 h.
5. Cells were subjected to 24 h of LBP pre-treatment at different contents (100 and 400 µg/mL). Then the medium was added with PA at 200 µg/mL for 12-h incubation, and later MC3T3-E1 cells were subjected to 48 h of miR-200b-3p inhibitors co-transfection with small interfering *Chrdl1* (si*Chrdl1*).

### Cell transfection

The cell transfection was performed as described. miR-200b-3p mimics/inhibitors and their negative control, and *Chrdl1* small interfering RNA (siRNA) and a small interfering negative control (siNC) were provided by Gene Pharma Co. Ltd (China). MC3T3-E1 cells were subjected to transfection with miR-200b-3p mimics/inhibitors/NC (50 pmol/mL), and si*Chrdl1* or siNC was transfected by Lipofectamine 2000 (Invitrogen) according to manufacturer’s protocols.

### Cell viability assay

Viability of cells was evaluated through Cell Counting Kit-8 (CCK-8) (Tongren, Shanghai, China). In each well of a 96-well plate, 4 × 10^3^ cells were grown, followed by 24-h of incubation. At 1 h prior to the completion of incubation, each well was added with CCK-8 reagent. Then optical density (OD) at 450 nm was detected for each well using the microplate reader.

### Dual-luciferase reporter gene assay

*Chrdl1* mRNA 3′UTR that contained wild-type (wt) or mutant (mut) sequence within candidate binding site of miR-200b-3p was amplified before cloning to miRNA-Report luciferase reporter vector (Shanghai Gene Chem Co. Ltd., China). Then, luciferase reporter gene assay was conducted according to a previous method. Briefly, MC3T3-E1 cells were plated to 24-well plates 24 h before mutant type (*Chrdl1-mut*) or wild type (*Chrdl1-wt*) luciferase vector co-transfected with miR-200b-3p mimics/inhibitors/NC through Lipofectamine 2000 (Invitrogen; Thermo Fisher Scientific Inc.). The luciferase activities were determined 24 h following transfection by Promega Dual-Luciferase Reporter Assay System (Promega Corp., Madison, WI, USA).

### Flow cytometry assay

The Annexin V-FITC apoptosis detection kit (Nanjing KeyGen Biotech Co., Ltd., Nanjing, China) was used to detect apoptotic rate using flow cytometer in accordance with manufacturer protocols. In brief, cells were subjected to 24-h incubation before they were harvested, followed by ice-cold phosphate buffer solution (PBS) washing, binding buffer (500 µL) re-suspending, and addition of Annexin V stock solution (5 µL) for 10 min of incubation at 4°C. Later, 5-µL propidium iodide (PI) was added to cells, followed by immediate analysis by FACSCalibur flow cytometer (BD Biosciences, San Jose, CA, USA).

### RT-PCR assay

TRIzol reagent (Takara Biotechnology Co. Ltd., Dalian, China) was used to extract total RNA from cells in all groups. Afterwards, the PrimeScript RT reagent kit equipped with gDNA Eraser (Takara Biotechnology Co., Ltd.) was utilized to synthesize cDNA through reverse transcription of RNA. The ABI 7500 Fast thermocycler (Applied Biosystems, Foster City, CA, USA) was employed for PCR amplification using SYBR-Green PCR kit (TransGen Biotech Co., Ltd., Beijing, China). Following were the PCR amplification conditions: 10 min at 95°C, then 15 s at 95°C for 40 cycles, and 45 s at 60°C for annealing/extension. Shanghai Sangon (Shanghai, China) was responsible for primer design. The sequences of specific primers for all genes are as follows: for miR-200b-3p: *5′-TAATACTGCCTGGTAATGATG-3*′ and *5′-CTCAACTGGTGTCGTGGA-3′*; for miR-200c-3p: *5′-TAATACTGCCGGGTAATGATGG-3′* and *5′-CCTCAACTGGTGTCGTGGA-3′*; for miR-429-3p: *5*′*-TAATACTGTCTGGTAATGCCG-3*′ and *5*′*-CTCAACTGGTGTCGTGGA-3*′, and for U6: *5*′*-CTCGCTTCGGCAGCACA-3*′ and *5*′*-AACGCTTCACGAATTTGCGT-3*′. Typically, gene expression was normalized relative to U6 and quantified by the 2^−∆∆Ct^ = [(C_T_gene of interest-C_T_internal control)sample A-(C_T_gene of interest-C_T_internal control)sample B].

### Western blotting

Cells were collected in each group, rinsed twice with PBS, and lysed with cold radioimmunoprecipitation assay buffer (RIPA buffer; Beyotime, Shanghai, China) supplemented with freshly prepared protease inhibitor cocktail (0.01%; Sigma, Shanghai, China). The cells were subjected to 30 min of incubation in ice. Then cell lysate was subjected to 10 min of centrifugation at 13,000 × g under 4°C to collect supernatants, which were (20–30-µg protein) isolated through 10% SDS-PAGE gel, followed by electrophoretic transfer onto the polyvinylidene fluoride (PVDF) membrane (Merck Millipore, Shanghai, China). Later, 5% skimmed milk was used to block protein blots, which were incubated with anti-GAPDH, anti-PPARγ, anti-*Chrdl1* antibodies (Beyotime), followed by incubation with goat anti-rabbit or anti-mouse secondary antibody (Beyotime). The blots were observed through enhanced chemiluminescence (ECL; Thermo Fisher Scientific Inc., Shanghai, China).

### Statistical analysis

SPSS 19.0 (IBM SPSS Statistics, USA) was used to assess each variable. Numerical data were expressed as mean ± standard deviation (SD) and differences were detected by Student’s t-test. Statistical analyses were performed through one-way analysis of variance (ANOVA) and Dunnett’s test in succession. A two-sided difference at *P* < 0.05 was deemed to be significant statistical.

## Results

### DEGs identification

**Fig. 1:**
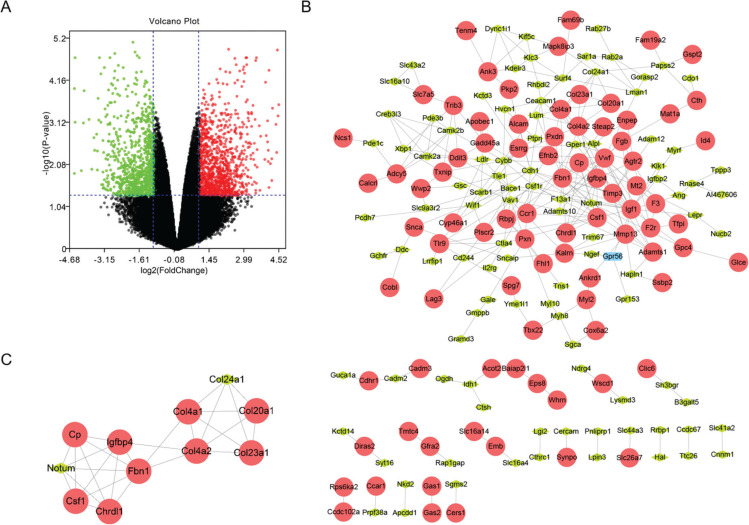
DEGs identification, PPI network construction and module analysis. (a) Volcano plot of DE-mRNAs from *GSE37676* data sets, the red and green dots represent the upregulation and downregulation (logFC >1.5 or < –1.5). (b) The PPI network of DEGs from *GSE37676* set was constructed using Cytoscape. (c) Significant module was obtained from PPI network. The color of node in the PPI network reflects the log (FC) value of the Z-score of gene expression, and the size of node indicates the number of interacting proteins with designated protein.

mRNA expression profile datasets of MC3T3-E1 cells treated with/without ascorbic acid contained expression profile matrix from 45,102 gene probes. A total of 4,072 DEGs were identified by GEO2R, including 458 downregulated and 347 upregulated DEGs (*P* < 0.05, logFC > 1.5 or < –1.5). The DEGs are displayed in Fig. 1a.

### PPI network construction and module analysis

All top 300 DEGs were mapped with STRING database for constructing the PPI network. Protein pairs having an integrated score of > 0.4 were screened. PPI network containing 288 edges was displayed with Cytoscape (Fig. 1b). Thereafter, MCODE, the plug-in used to search optimized scoring parameters to generate optimal network results, was utilized to search for modules from the network. Module 1 consisted of 28 edges and 11 nodes and had a higher score over other modules (Fig. 1c). *Chrdl1* is a key gene from the most significant module.

### Network of Chrdl1 co-expression

**Fig. 2:**
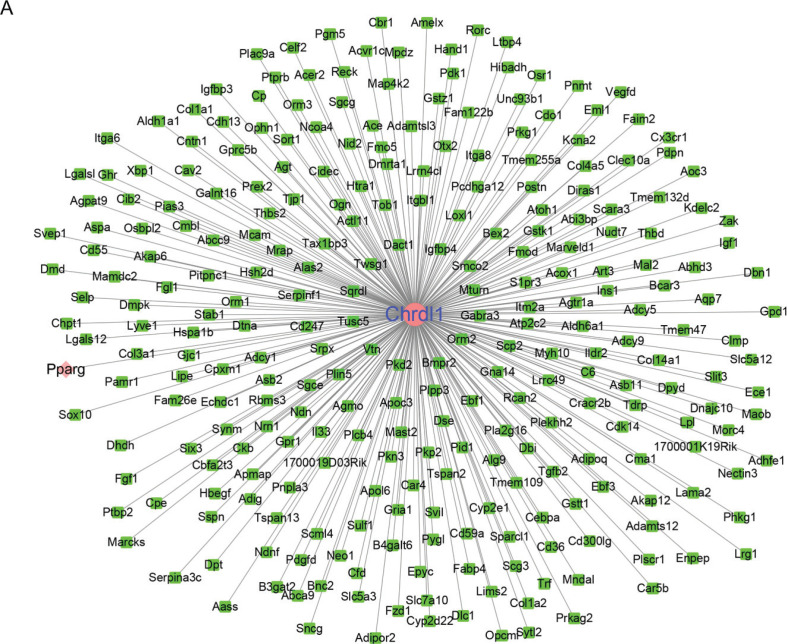
Network of *Chrdl1* co-expression. (a) COEXPEDIA database was used to construct the co-expression network of *Chrdl1*.

Gene co-expression that showed functional association with *Chrdl1* was estimated by COEXPEDIA database to examine the effect and molecular mechanism of *Chrdl1* on disease. *Chrdl1* had a co-expression with 274 genes (Fig. 2). Interestingly, *Chrdl1* was co-expressed with PPARγ.

### Different concentrations of PA significantly regulated the mRNA expression levels of Chrdl1, PPARγ, and miR-200b-3p/200c-3p/429-3p

**Fig. 3:**
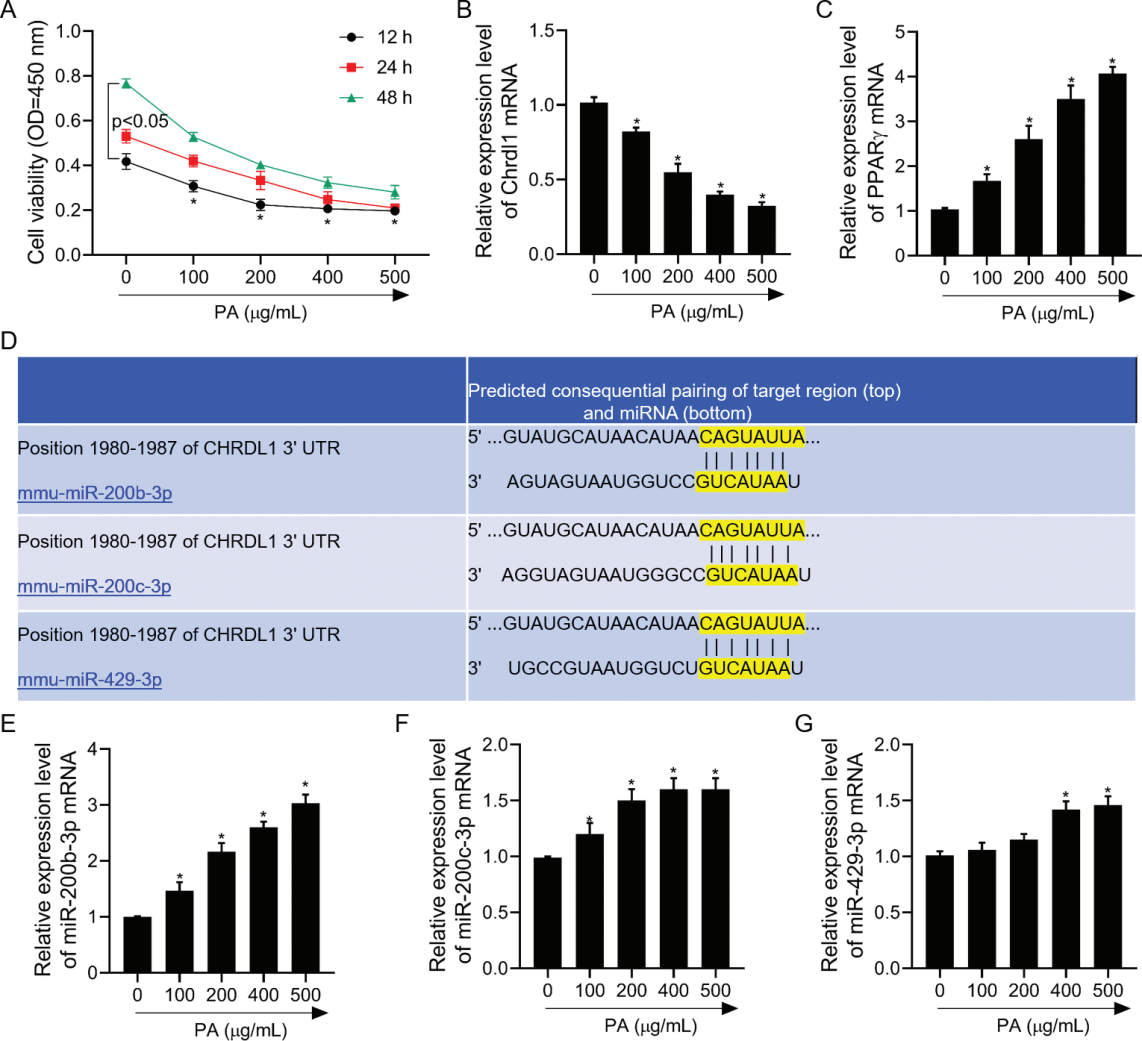
PA-regulated cell viability and the mRNA levels of *Chrdl1*, PPARγ, miR-200b-3p, miR-200c-3p, and miR-429-3p. After MC3T3-E1 cells were treated with PA (0, 100, 200, 400, and 500 μg/mL), (a) CCK-8 assay was used to detect cell viability; (b) RT-PCR assay was used to detect *Chrdl1* mRNA level; (c) RT-PCR assay was used to detect PPARγ mRNA level; (d) alignment of one predicted miR-200b-3p, miR-200c-3p, and miR-429-3p target site in the *Chrdl1-3’-UTR*; (e) RT-PCR assay was used to detect miR-200b-3p mRNA level; (f) RT-PCR assay was used to detect miR-200c-3p mRNA level; and (g) RT-PCR assay was used to detect miR-429-3p mRNA level. GAPDH and U6 were used as load control. Data are presented as mean ± SD. ^**^P < 0.01 and ^*^P < 0.05 versus Con. group.

The viability of cells was significantly decreased after 12/24/48 h of PA treatment at 500, 400, 200, 100, and 0 μg/mL with MC3T3-E1 cells (Fig. 3a). Next, cells treated with PA at 200 μg/mL for 12 h were used to perform follow studies. Chrdl1 and PPARγ mRNA expression decreased depending on dose (Figs. 3b and 3c). Furthermore, we found that Chrdl1 was the miR-426-3p, miR-200c-3p, and miR-200b-3p target gene of TargetScan database (Fig. 3d). This study found that PA significantly increased miR-426-3p mRNA, miR-200c-3p, and miR-200b-3p expressions, which presented dosage-dependency relations (Figs 3e–3g). The amplification multiples of miR-200b-3p mRNA were higher than that of miR-200c-3p/429-3p by RT-PCR assay. Therefore, miR-200b-3p was used to perform follow studies.

### LBP enhanced the viability of MC3T3-E1 cells under PA treatment and Chrdl1 was recognized as specific miR-200b-3p target gene

**Fig. 4:**
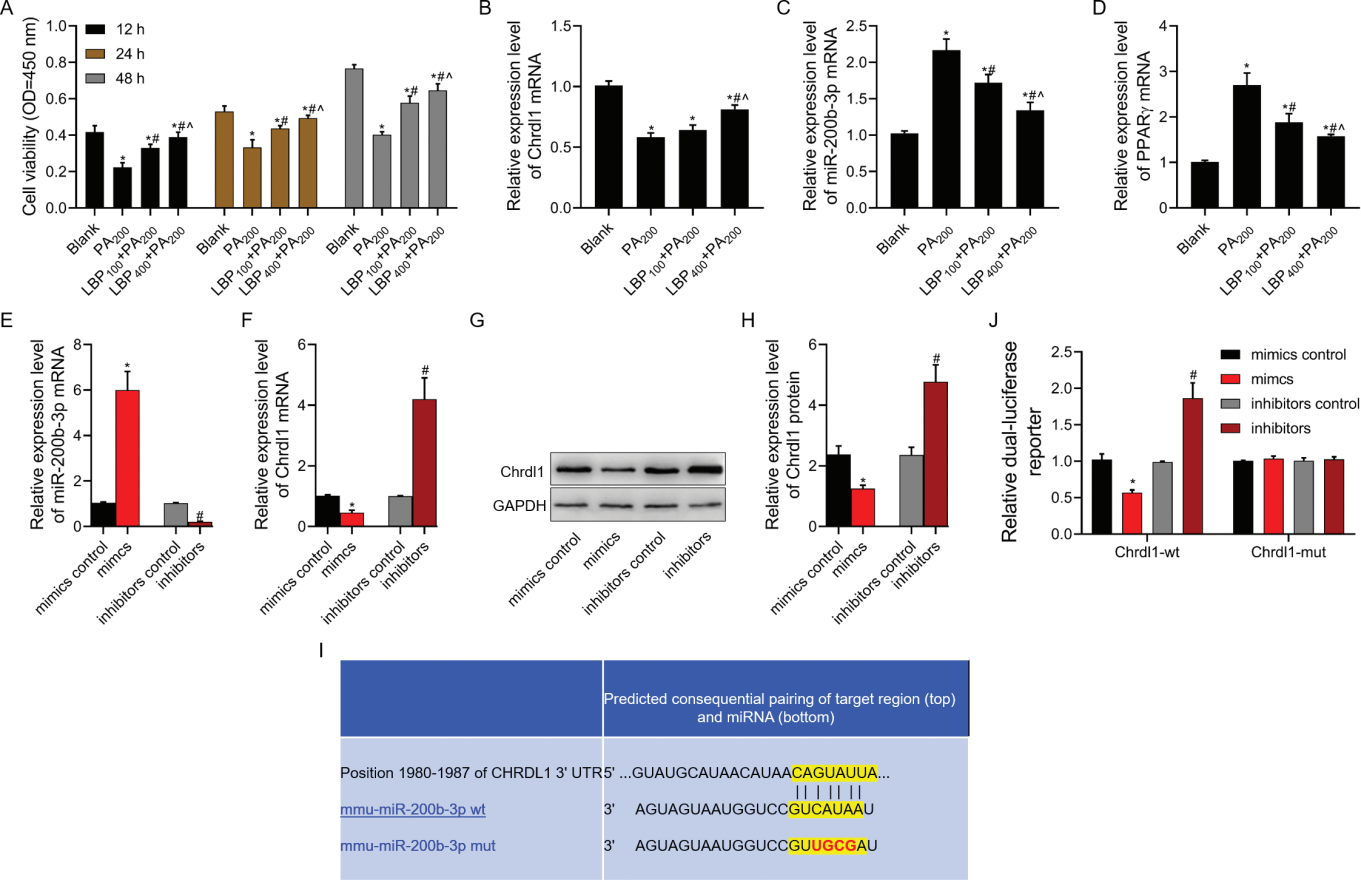
LBP enhanced the viability of MC3T3-E1 cells under PA treatment and *Chrdl1* was recognized as the specific miR-200b-3p target gene. After cells were pre-treated with 100 or 400 μg/mL of LBP, (a) CCK-8 assay was used to detect cell viability in cells treated with PA; (b) RT-PCR assay was used to detect *Chrdl1* mRNA level in cells treated with PA; (c) RT-PCR assay was used to detect miR-200b-3p mRNA level in cells treated with PA; (d) RT-PCR assay was used to detect PPARγ mRNA level in cells treated with PA after cells were transfected with miR-200b-3p mimics and miR-200b-3p inhibitors; (e) RT-PCR assay was used to detect miR-200b-3p mRNA level; (f) RT-PCR assay was used to detect *Chrdl1* mRNA level; (g, h) western blot assay was used to detect *Chrdl1* protein level; (i) *Chrdl1* interacted with miR-200b-3p, and *Chrdl1*-mt and *Chrdl1*-wt binding sites were displayed by TargetScan database; and (j) luciferase report system was used to detect the targeting miR-200b-3p with *Chrdl1*. GAPDH was used as a load control. Data are presented as mean ± SD. ^*^P < 0.05 versus blank group/or mimics negative control group, ^#^*P* < 0.05 versus PA200 group or inhibitors negative control group, and ^^^*P* < 0.05 versus LBP100 + PA200 group.

As shown in Fig. 4a, 100 or 400 μg/mL of LBP markedly increased MC3T3-E1 cell viability under PA treatment by CCK-8 assay. Meanwhile, the function of PA decreasing the *Chrdl1* mRNA level, and increasing miR-200b-3p and PPARγ mRNA levels was partly reversed by treatment with 100 or 400 μg/mL of LBP (Figs 4b–4d). After miR-200b-3p mimics/inhibitors and their negative controls were transfected into MC3T3-E1 cells, *Chrdl1* protein and mRNA levels were markedly downregulated, and *Chrdl1* protein and mRNA expression were remarkably upregulated (Figs 4e–4h). *Chrdl1* interacted with miR-200b-3p, and *Chrdl1*-mut and *Chrdl1*-wt binding sites were displayed (Fig. 4i). According to results of luciferase reporter gene assay, miR-200b-3p mimics co-transfected with *Chrdl1*-wt evidently lowered the luciferase activities. In addition, miR-200b-3p inhibitors co-transfected with *Chrdl1*-wt markedly increased the luciferase activities. Meanwhile, miR-370-3p mimics or inhibitors co-transfected with *Chrdl1*-mut made no difference to the luciferase activities of MC3T3-E1 cells (Fig. 4j).

### LBP inhibited PA-mediated apoptosis in MC3T3-E1 cells under miR-200b-3p mimics transfection

**Fig. 5:**
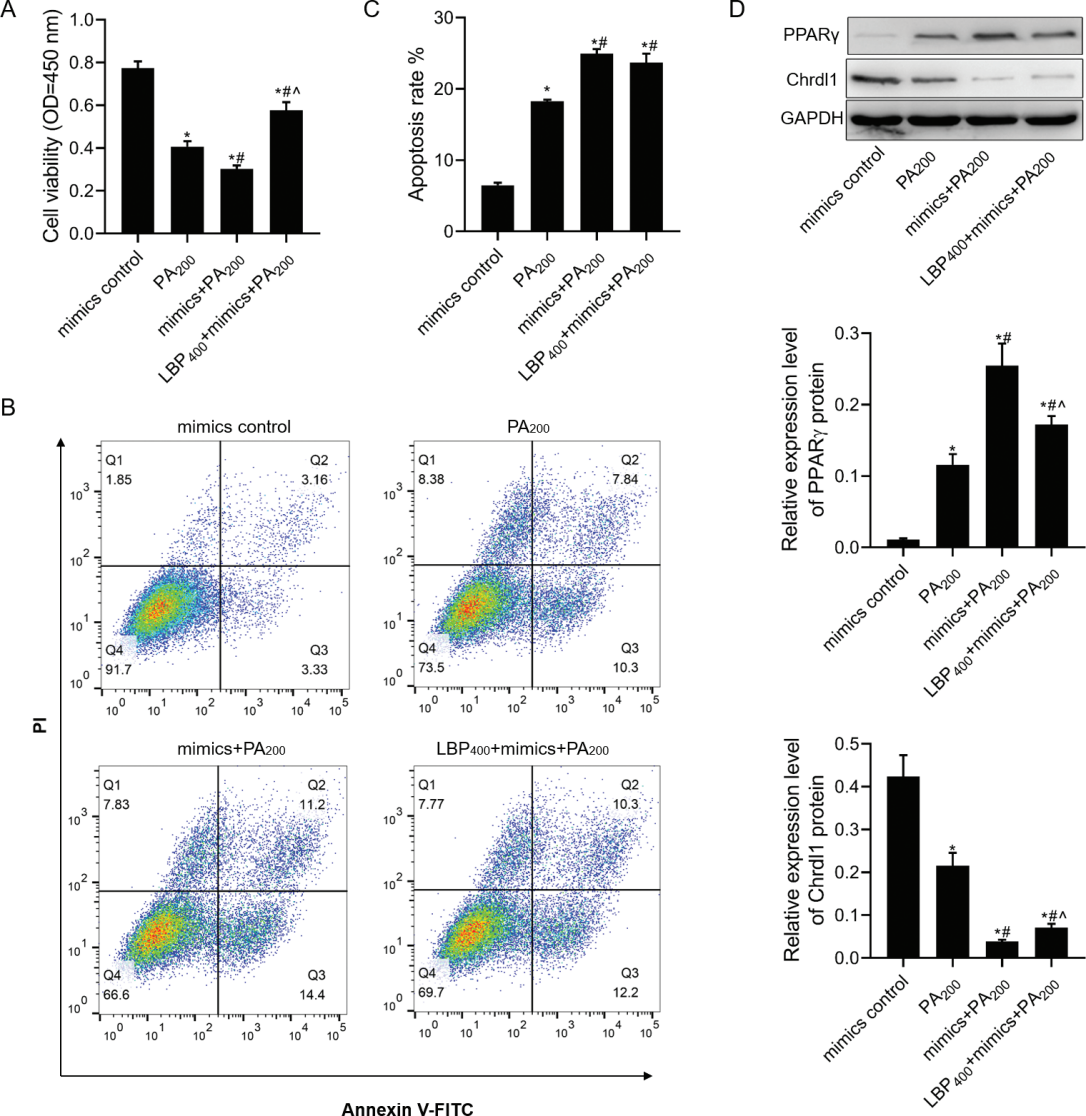
LBP inhibited PA-mediated apoptosis in MC3T3-E1 cells transfected with miR-200b-3p mimics. After cells transfected with miR-200b-3p, mimics were treated with LBP and PA: (a) CCK-8 assay was used to detect cell viability; (b, c) flow cytometry was used to detect cell apoptosis; (d) western blot assay was used to detect PPARγ and *Chrdl1* protein levels. GAPDH was used as a load control. Data are presented as mean ± SD. ^*^P < 0.05 versus mimics negative control group, ^#^*P* < 0.05 versus PA200 group, and ^^^*P* < 0.05 versus mimics+PA200 group.

For further studying LBP action mechanism in affecting PA-induced MC3T3-E1 cell apoptosis, flow cytometry (FCM) and CCK-8 assays were carried out to determine the viability and apoptosis of MC3T3-E1 cells with different treatments. According to our findings, miR-200b-3p mimics could inhibit MC3T3-E1 cell viability under PA treatment and further aggravate PA-induced cell apoptosis (Figs 5a–5c). Next, the function of miR-200b-3p mimics to some extent lessened cell viability while promoted cell apoptosis treatment with PA by treatment with 400 μg/mL of LBP. In addition, miR-200b-3p further increased PA-medicated PPARγ expression and decreased PA-medicated *Chrdl1* expression (Fig. 5d). Meanwhile, the function of miR-200b-3p mimics increasing PPARγ protein level and decreasing *Chrdl1* protein level of MC3T3-E1 cells treated with PA was reversed to some extent by treatment with 400 μg/mL of LBP.

### LBP further inhibited PA-mediated apoptosis of MC3T3-E1 cells under miR-200b-3p inhibitor transfection

**Fig. 6:**
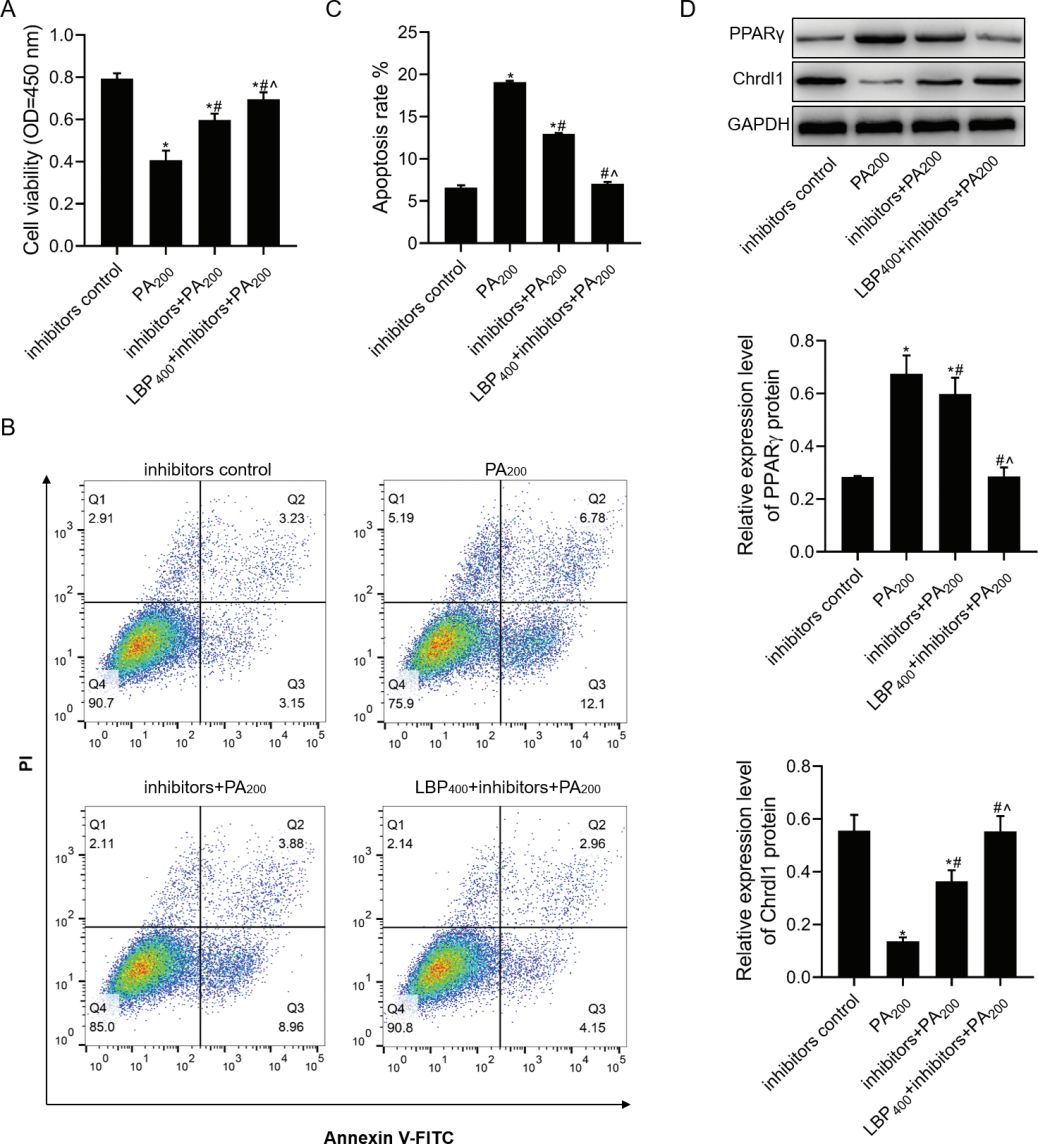
LBP further inhibited PA-mediated apoptosis in MC3T3-E1 cells transfected with miR-200b-3p inhibitors. After cells transfected with miR-200b-3p, inhibitors were treated with LBP and PA: (a) CCK-8 assay was used to detect cell viability; (b,c) flow cytometry was used to detect cell apoptosis; (d) western blot assay was used to detect PPARγ and *Chrdl1* protein levels. GAPDH was used as a load control. Data are presented as the mean ± SD. ^*^P < 0.05 versus inhibitors negative control group, ^#^*P* < 0.05 versus PA200 group, and ^^^*P* < 0.05 versus inhibitors+PA200 group.

Based on the above-mentioned results, miR-200b-3p inhibited the PA-mediated apoptosis of LBP-treated MC3T3-E1 cells. According to our results, miR-200b-3p inhibitors could upregulate MC3T3-E1 cell viability under PA treatment and inhibit PA-induced cell apoptosis (Figs 6a–6c). Next, the function of miR-200b-3p inhibitors to some extent increased MC3T3-E1 cell viability and decreased their apoptosis under PA treatment with 400 μg/mL of LBP. In addition, miR-200b-3p inhibition decreased PA-medicated PPARγ expression and increased PA-medicated *Chrdl1* expression (Fig. 6d). Meanwhile, the function of miR-200b-3p inhibitors decreasing PPARγ protein level and increasing *Chrdl1* protein level of MC3T3-E1 cells treated with PA was reversed to some extent by treatment with 400 μg/mL of LBP.

### LBP affected PA-mediated apoptosis by regulating miR-200b-3p/Chrdl1/PPARγ

**Fig. 7:**
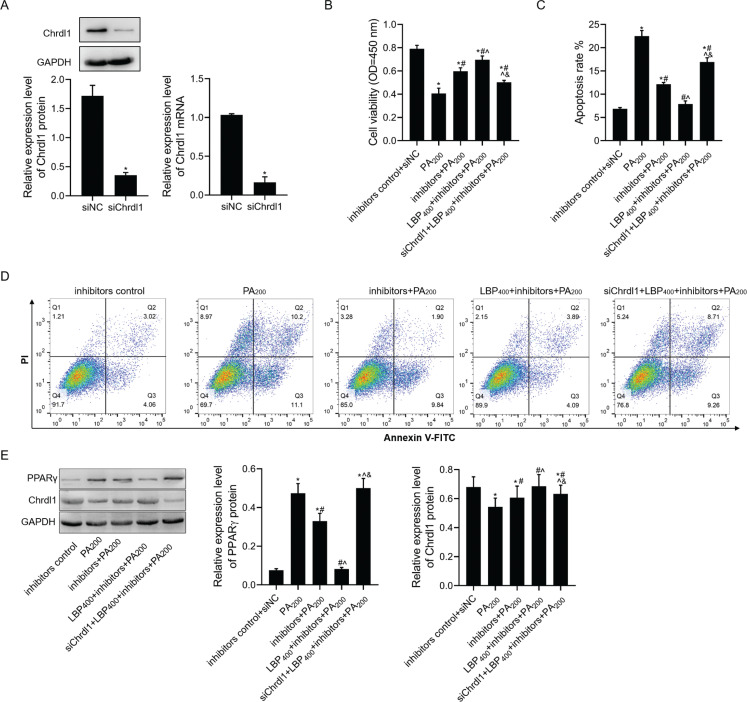
LBP affected PA-mediated apoptosis by regulating miR-200b-3p/*Chrdl1*/PPARγ. (a) Western blot and RT-PCR assays were used to detect the protein and mRNA levels of *Chrdl1* in cells transfected with si*Chrdl1* after cells were transfected with inhibitors and si*Chrdl1* was treated with LBP and PA; (b) CCK-8 assay was used to detect cell viability; (c,d) flow cytometry was used to detect cell apoptosis; (e) western blot assay was used to detect PPARγ and *Chrdl1* protein levels. GAPDH was used as a load control. Data are presented as mean ± SD. ^*^P < 0.05 versus inhibitors control+siNC group, ^#^*P* < 0.05 versus PA200 group, ^^^*P* < 0.05 versus inhibitors+PA200 group, and ^&^*P* < 0.05 versus LBP400+inhibitors+PA200 group.

First, *Chrdl1* protein and mRNA expression were notably downregulated among MC3T3-E1 cells under siRNA-*Chrdl1* transfection (Fig. 7a). After miR-200b-3p inhibitors/siRNA-*Chrdl1* co-transfection in MC3T3-E1 cells, LBP effects on increasing the viability of MC3T3-E1 cells but reducing their apoptosis were abolished partly within PA-treated cells (Figs 7b–7d). Furthermore, LBP inhibited PPARγ protein level and increased *Chrdl1* protein level in MC3T3-E1 cells treatment with PA by inhibiting miR-200b-3p expression. However, the function of LBP regulating PPARγ and *Chrdl1* protein levels in MC3T3-E1 cells treatment with PA were abolished by co-transfection with miR-200b-3p inhibitors and siRNA-*Chrdl1* (Fig. 7e).

## Discussion

The obesity-induced osteoporosis has always been our research focus. LBP can improve obesity and increase BMD, and its clinical effects have been approved by evidence-based medicine (22). However, similar to many Chinese patent medicines, its precise effect has not been recognized by modern medicine. This would provide new thinking and method for the future clinical treatment and new drug development if its major action pathway could be determined at molecular level. Our research group had previously used PA to treat osteoblasts so as to simulate the cellular environment of obesity-induced osteoporosis, and we discovered that LBP could suppress endoplasmic reticulum (ER) stress to reduce the PA-induced osteoblast apoptosis (11). Nonetheless, this was only the preliminary exploration. More intensive studies are needed to examine whether LBP could alleviate obesity to improve osteoporosis. We utilized bioinformatics to analyze *GSE37676* chip data and confirmed that *Chrdl1* was the key gene of the most significant module in the PPI network, which was markedly upregulated after AA treatment. The bone morphogenetic protein (BMP) antagonist *Chrdl1* is the target of miR-200a family and involved in osteoclast differentiation (23). *Chrdl1* can hinder the BMP-mediated enhanced invasion and migration of breast cancer cells (24). Hypermethylation of *Chrdl1* promoter induces gastric cell proliferation (25). Based on the above results, we predicted that *Chrdl1* can have a vital role during the apoptosis of osteoblasts. Consequently, the Co database was used to predict the *Chrdl1* co-expression network. Intriguingly, *Chrdl1* was co-expressed with PPARγ. PPARγ is an important regulatory factor during the differentiation process of pre-adipocytes, and its activation could accelerate adipocyte differentiation, thus aggravating obesity in patients. According to reports, LBP can reduce PPARγ activity to relieve obesity (9, 10). We speculated based on the above research results that *Chrdl1* was closely correlated with the obesity-induced osteoporosis. RT-PCR experimental results verified that PA could evidently downregulate *Chrdl1* expression depending on the dose; meanwhile, PPARγ mRNA level was notably upregulated with increase in PA concentration, revealing that the PA-induced osteoblast apoptosis could be achieved through regulating the expression of *Chrdl1* and PPARγ.

As it is well known, miRNAs can specifically regulate one or more target genes involved in genesis and development of numerous diseases and participate in regulating cell functions. Different miRNAs have exerted vital roles in obesity and osteoporosis (12, 14, 16, 20). *Chrdl1* has been recognized to be the miR-200b-3p target gene using the TargetScan database. According to our findings, miR-200b-3p was upregulated among MC3T3-E1 cells with increasing PA concentration. Next, after MC3T3-E1 cells were incurred to pre-treatment with 100 or 400 μg/mL of LBP, cell viability increased significantly among PA-treated MC3T3-E1 cells. Based on our results, LBP significantly increased PA-mediated decrease in *Chrdl1* mRNA levels and inhibited PA-mediated increase in miR-200b-3p and PPARγ mRNA levels. It suggested that LBP could regulate the miR-200b-3p/*Chrdl1*/PPARγ axis to exert a series of functions in PA-treated cells. To further verify the above-mentioned results, CCK-8, flow cytometry, and western blotting were used to detect viability, apoptosis as well as PPARγ and *Chrdl1* expression in miR-200b-3p-transfected cells. Overexpression of miR-200b-3p aggravated PA-induced apoptosis and further increased PA-mediated increase in PPARγ protein level and decrease in PA-mediated inhibition in *Chrdl1* protein level. Meanwhile, the function of PA affected cell proliferation, apoptosis, and the expression of PPARγ and *Chrdl1* proteins by LBP treatment. miR-200b-3p inhibition had opposite effect relative to that of miR-200b-3p overexpression in MC3T3-E1 cells co-treatment of PA with LBP.

These results proved that LBP could downregulate miR-200b-3p expression and suppress the PA-induced cell apoptosis; meanwhile, it could promote *Chrdl1* expression and indirectly suppress PPARγ expression. When si*Chrdl1* and inhibitors were co-transfected into cells, the suppression of LBP on PA-induced cell apoptosis was also suppressed partially. This was because the PA-induced cell apoptosis was not only realized through upregulating miR-200b-3p but also by other molecules to exert pro-apoptotic effect. Nonetheless, it is certain that suppressing *Chrdl1* and miR-200b-3p expression at the same time could evidently weaken the anti-apoptotic effect of LBP on PA-treated cells. Meanwhile, we also discovered that PPARγ expression in cells co-transfected with si*Chrdl1* and inhibitors displayed no significant difference compared with that in PA-treated cells.

## Conclusion

PA can partially upregulate miR-200b-3p and PPARγ expressions to specifically suppress *Chrdl1* expression and promote osteoblast apoptosis. LBP can suppress the PA-induced osteoblast apoptosis through the miR-200b-3p/*Chrdl1*/PPARγ axis, which has also verified that LBP shows certain protection on the obesity-induced osteoporosis.

## Conflict of interest and funding

The authors declare that they have no conflicts of interest. The project was supported by the Ningbo City Government Foundation of China (No. 2018A610260).
